# Comprehensive Investigation of a Novel Schiff Base: Synthesis, Anticancer Efficacy, Gene Expression Profiling, and Computational Analyses

**DOI:** 10.3390/ph19020332

**Published:** 2026-02-18

**Authors:** Tugba Agbektas, Özhan Pazarcı, Ayca Tas, Alakbar Huseynzada, Ruslan Guliyev, Ulviyya Hasanova, Emre Can Buluz, Savas Kaya, Alejandro Morales-Bayuelo, Yavuz Silig

**Affiliations:** 1 Department of Food Processing Technologies Services, Yıldızeli Vocational School, Sivas Cumhuriyet University, Sivas 13001, Türkiye; tubaagbektas@cumhuriyet.edu.tr; 2 Department of Orthopedics and Traumatology, Adana City Training and Research Hospital, Adana 01000, Türkiye; drpazarci@gmail.com; 3 Department of Biochemistry, Faculty of Medicine, Sivas Cumhuriyet University, Sivas 58140, Türkiye; aycatas@cumhuriyet.edu.tr (A.T.); ysilig@cumhuriyet.edu.tr (Y.S.); 4 ICRL, Baku State University, Z. Khalilov 23, Baku AZ1148, Azerbaijan; alakbar.huseynzada1117@gmail.com; 5 GPOGC SRI, Azerbaijan State Oil and Industry University, Baku AZ1148, Azerbaijan; ruslandjan01@gmail.com; 6 ICESCO Biomedical Materials Department, Baku State University, Z. Khalilov 33, Baku AZ1148, Azerbaijan; u.alimammad@gmail.com; 7 Department of Biotechnology, Institute of Science, Ege University, Izmir 35100, Türkiye; emrecn-35@hotmail.com; 8 Department of Chemistry, Faculty of Science, Sivas Cumhuriyet University, Sivas 58140, Türkiye; 9 Grupo Genoma, Escuela de Medicina, Universidad del Sinú, Cartagena 130001, Colombia; amorales@unisinucartagena.edu.co

**Keywords:** SAOS-2, anticancer drug, gene expression, DFT, molecular docking, magnetism analyses

## Abstract

(1) **Background:** This study evaluates the anticancer potential of a newly synthesized azomethine-based compound, 6,6′,5,8-Dioxa-2,11-diazadodeca-1,11-diene-1,12-diyl)bis(4-bromo-2-methoxyphenol) (B-134-0), against osteosarcoma (SAOS-2) cells, focusing on its effects on apoptosis and DNA-damage-related gene expression. (2) **Methods:** B-134-0 was synthesized via condensation and tested at eight concentrations (0.5–100 μg/mL) for 24, 48, and 72 h. Cytotoxicity was assessed through MTT assay, and gene expression levels of *TP53*, *RAD51*, *BRCA2*, *CASP2*, *MYC, MDM2*, *CDKN1A*, *ERCC1*, *ATR*, and *PRKDC* were quantified through qPCR using the ΔΔ_Ct_ method. Molecular docking and DFT analyses were performed to explore structural stability and protein interactions. (3) **Results:** B-134-0 exhibited strong time-dependent cytotoxicity (IC_50_: 71.58, 54.36, and 12.59 μg/mL at 24, 48, and 72 h, respectively) and significantly modulated the expression of cell cycle and DNA-repair-associated genes. The compound notably downregulated *TP53*, *RAD51*, *CASP2*, *MYC*, and *MDM2*, while *CDKN1A* and *BRCA2* showed relative upregulation, indicating activation of the DNA damage response. Docking results revealed strong binding affinity with *BRCA2* and *CDKN1A*, consistent with experimental findings. (4) **Conclusions:** These results indicate that B-134-0 exhibits potent anticancer activity by modulating DDR and apoptosis pathways, with strong molecular stability, suggesting its promise as a therapeutic candidate for osteosarcoma.

## 1. Introduction

Osteosarcoma is rare cancer and the most frequent primary malignant bone tumor. Though it is uncommon, the disease is more prevalent among teenagers and young adults. If untreated, osteosarcoma can be fatal. Despite modern treatment protocols, including combined chemotherapy, surgery, and sometimes radiotherapy, the ten-year survival rate ranges between 48.9% and 59.8% [1]. It is of paramount importance to develop a greater understanding of the underlying mechanisms behind tumor advancement and metastasis to pinpoint and create more efficient remedies. Cancer cell lines and tissue samples serve as vital tools to examine these mechanisms and appraise novel targeted therapies [2]. Certain physical, chemical, and biological agents are believed to contribute to osteosarcoma carcinogenesis. Among these, ultraviolet and ionizing radiation are the most widely recognized [3]. Numerous therapeutic agents have been developed for cancer treatment; however, despite their efficacy against cancer cells, severe side effects, such as nephrotoxicity, neurotoxicity, and ototoxicity, have prompted the search for novel compounds with improved safety profiles. In this context, Schiff-base-derived metal complexes have gained considerable attention, with reported applications in drug design, analytical chemistry [4], industrial processes [5,6], and antimicrobial fields [7]. Newly synthesized azomethine-containing compounds, in particular, have emerged as promising candidates for cancer therapy due to their enhanced efficacy and selectivity [8,9,10]. Recent studies have highlighted the potential of Schiff base and azomethine compounds to act as anticancer agents by disrupting cellular redox balance and interfering with DNA damage response (DDR) mechanisms, thereby inducing apoptosis and growth inhibition in tumor cells. Azo azomethine derivatives, characterized by the chromophoric –N=N– and –CH=N– groups, have likewise attracted significant interest. Azo dyes encompass various subclasses, including amido-azo, oxy-azo, diazo, tetrazo, and other polyazo derivatives [11]. Beyond their industrial use, many azo compounds function as chromogenic reagents in colorimetric assays and as auxiliary agents in complexometric titrations. Furthermore, certain azo derivatives have been reported to display potent antimicrobial properties [12]. Schiff bases have also been widely studied and shown to exhibit a broad spectrum of biological activities, including antitumor, antibacterial, fungicidal, and anticarcinogenic effects [13,14]. Accordingly, Schiff base derivatives have been extensively synthesized and investigated as potential therapeutic agents in a variety of diseases, including malaria, viral infections, diabetes, fungal infections, inflammatory disorders, corrosion-related conditions, cancer [15], and HIV [16,17,18,19]. For instance, studies conducted on breast (MCF-7), liver (HepG2), lung (A549), and colorectal (HCT116) cancer cell lines revealed that compounds acting through tyrosine kinase (TRK) inhibition exhibit significant antiproliferative and anticancer activities [20]. Noser et al. (2021) demonstrated that quinazoline and indole amino acid Schiff bases exerted potent cytotoxic effects against breast cancer cells by promoting ROS-mediated inhibition of mitochondrial complex I-associated hexokinase, leading to AMPK activation, mTOR inhibition, p53 activation, and cell cycle arrest [21]. Similarly, Hu et al. (2024) showed that copper(II) Schiff base complexes increased intracellular ROS levels and disrupted the redox balance in MCF-7 cells, resulting in oxidative DNA damage, p53-linked apoptosis, and autophagy induction [22]. These findings emphasize that Schiff base compounds can modulate DDR pathways either through direct DNA interaction or indirectly via oxidative stress, ultimately leading to apoptosis and inhibition of tumor proliferation. Apoptosis is a genetically regulated process of cell death that contributes to development and homeostasis in normal tissues. Its regulation is critical for normal growth, homeostasis, development, and cancer treatment [23]. Tumor formation may occur even in cases where very few cells undergo apoptosis, irrespective of the proliferation rate. The delayed activation of apoptotic pathways is a vital phenomenon in osteosarcoma regression and therapy resistance [24]. Apoptosis evasion is both involved in cancer initiation and one of the distinctive features of human cancers, including osteosarcoma. The contemporary therapeutic regimens of cancer cells, that is, response to chemo-, radio-, or immunotherapy, are mainly attributed to cell death induction in tumorous cells, while resistance to apoptosis and therapy coexist for response to cytotoxic treatments [25]. The conversion of normal induction to apoptosis can cause the development of abnormal cells, uncontrollable cell division, and mutation accumulation. Therefore, regulating apoptosis is crucial in cancer therapy. Many human cancers have dysregulated cell cycle progression; therefore, cellular processes like proliferation, differentiation, and apoptosis may be influenced, resulting in tumorigenesis [26]. Drugs that increase or decrease unregulated cell proliferation have the potential to impede tumor growth and may be a potent treatment strategy for malignant osteosarcoma cancer [27].

The main aim of this article is to investigate the anticancer and gene expression effects of a newly synthesized Schiff base and to highlight its interactions and reactivity using DFT, molecular docking, and magnetism calculations.

## 2. Results

### 2.1. Experimental Findings on Anticancer Activity and Gene Expression

#### 2.1.1. Synthesis Part

**Figure 1:**
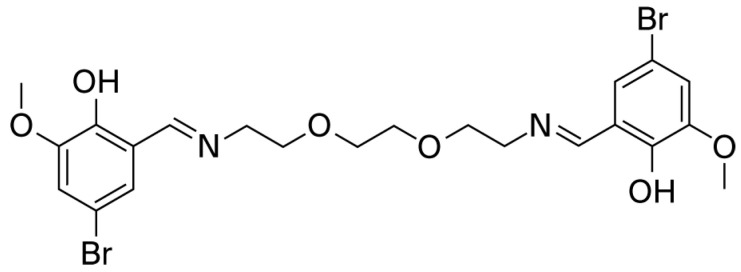
Compound B-134-0 containing azomethine group (6,6′,5,8-Dioxa-2,11-diazadodeca-1,11-diene-1,12-diyl)bis(4-bromo-2-methoxyphenol).

The target compounds were synthesized through straightforward condensation reactions between the selected aldehydes and amines. A notable advantage of this procedure is its simplicity, as the resulting solid products do not require further purification; gentle washing with a small volume of distilled water is sufficient. Another benefit lies in the fact that the reactions occur under non-catalytic conditions. Structural confirmation of the obtained products was achieved through 1H and 13C NMR spectroscopy. The absence of signals corresponding to the aldehyde functional group, along with the appearance of resonances attributed to the azomethine linkage, provided clear evidence for the successful formation of the designed compounds (Figure 1).

#### 2.1.2. In Vitro Assay for Cytotoxicity Activity (MTT Assay)

**Figure 2:**
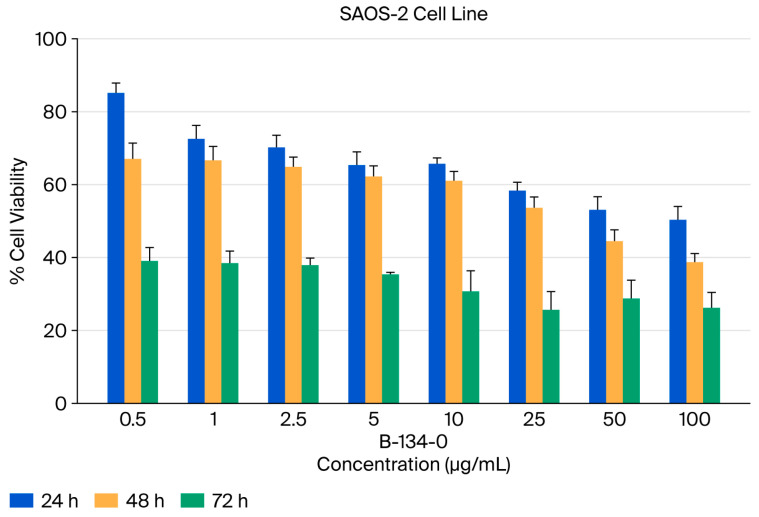
Viability test (MTT) of B-134-0 applied to SAOS-2 cells at the end of 24, 48, and 72 h.

**Figure 3:**
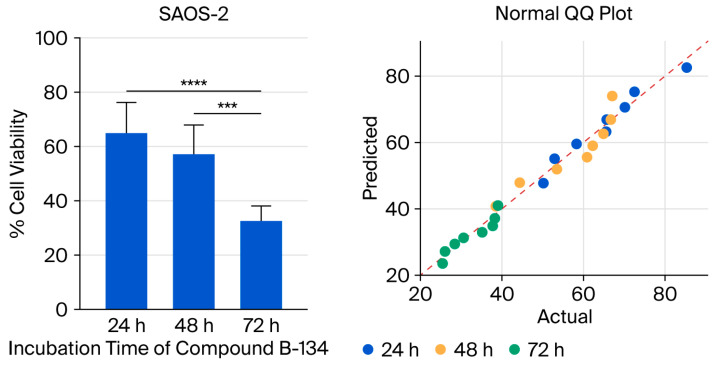
Comparison of the effect of B-134-0 compound on SAOS-2 cell viability at concentrations of 100–0.5 µg/mL after 24, 48, and 72 h of incubation and normal QQ plot analysis for SAOS-2 cells (**** *p* < 0.0001, *** 0.0001).

The aim of the study was to evaluate the viability of SAOS-2 cells treated with the B-134-0 compound by exploring the significance of different incubation periods, specifically 24 h, 48 h, and 72 h. The statistical analysis showed that B-134-0 had a significant impact on the SAOS-2 cell line across all three incubation periods (**** *p* < 0.05, *** *p* < 0.05), as shown in Figure 2. However, there was no significant relationship found (*p* > 0.05) when comparing the effects of 24 h and 48 h of incubation of B-134-0 in SAOS-2 cells. Significant relationships were found when comparing the 48 h and 72 h incubation periods of the B-134-0 compound in SAOS-2 cells (*** *p* = 0.0001) (Figure 3, Table 1). Multiple comparisons revealed a significant relationship between the B-134-0 compound incubation periods of 24 h and 72 h in SAOS-2 cells (**** *p* < 0.0001) (Figure 3, Table 1). The IC_50_ values of the compound over 24, 48, and 72 h of incubation were found to be 71.58 µg/mL, 54.36 µg/mL, and 12.59 µg/mL, respectively (Table 2). Therefore, upon examination of the incubation times of the B-134-0 compound on the SAOS-2 cell line, it is evident that it is most effective after 72 h.

The normal QQ plot indicates that the data follow a normal distribution. The proximity between the observed values and the expected quantiles of the theoretical normal distribution suggests that the data adhere to the assumption of normality. This finding supports the validity of the normality assumption and strengthens the robustness of subsequent statistical analyses. Additionally, the Shapiro–Wilk and Kolmogorov–Smirnov test results further confirm that the data from the SAOS-2 cell line follow a normal distribution, with *p*-values supporting the assumption of normality (Table 3).

#### 2.1.3. Cell Morphology Analysis

**Figure 4:**
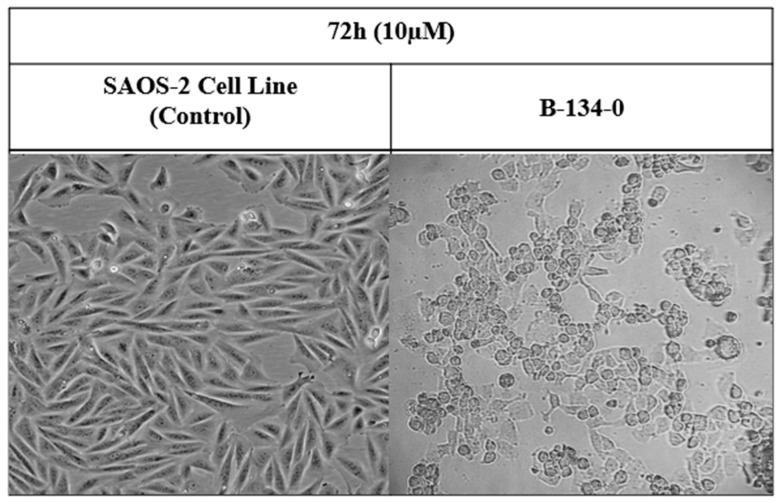
Morphological changes in SAOS-2 cells after 72 h of incubation with concentrations (10 μM) of B-134-0. The results presented are from experiments that were carried out and photographed microscopically.

When B-134-0 compounds were applied to each SAOS-2 cancer cell at a dosage of 1 g/mL, the morphology of the cancer cells changed compared to the control group (Figure 4).

#### 2.1.4. Bioinformatics Analysis

**Figure 5:**
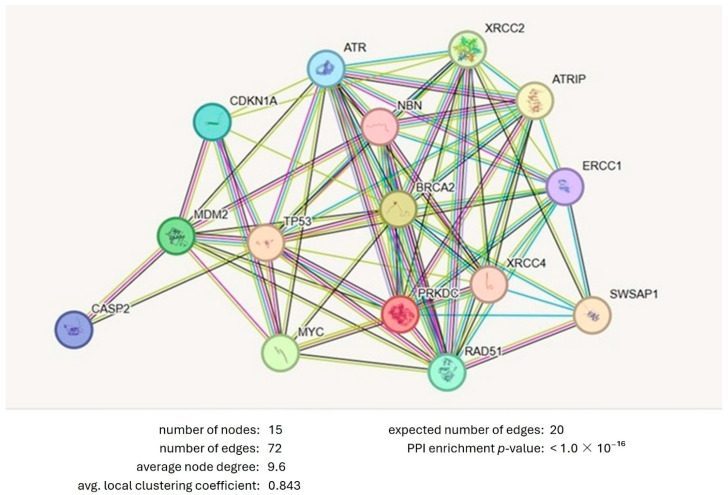
Interaction of proteins involved in the cell cycle and apoptosis with each other and other proteins.

**Figure 6:**
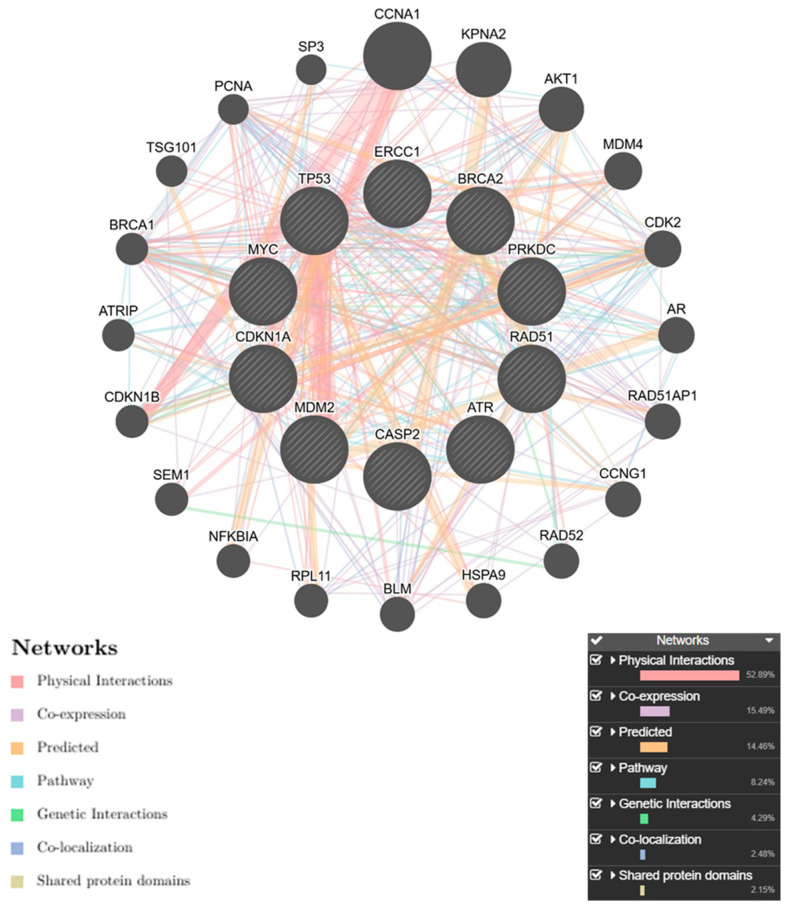
Gene interactions among *Tp53*, *RAD51*, *BRCA2*, *CASP2*, *MYC*, *MDM2*, *CDKN1A*, *ERCC1*, *ATR,* and *PRKDC* in SAOS-2 carcinoma. The color of the connecting line represents the type of interaction.

The study utilized the String v11 program to determine protein–protein interactions in the cell cycle and apoptosis pathways. The focus was on the interactions of Tp53, RAD51, BRCA2, CASP2, MYC, MDM2, CDKN1A, ERCC1, ATR, and PRKDC proteins involved in both cell cycle and apoptosis with themselves and other proteins. The findings are presented in Figure 5. Thirty-one proteins in the second shell were found to interact with fifteen proteins in the first shell, with homology scores ranging from 0.739 to 0.999 (Table 4, Figure 5). The PPI enrichment *p*-value was found to be 2.83 × 10^−11^. The analysis using GenMANIA software for GGI involving genes such as AKT1, RPL11, CCNG1, HSPA9, NFKBIA, SP3, CCNA1, MDM4, CDK2, AR, RAD52, CDKN1B, TSG101, KPNA2, PCNA, RAD51AP1, CDKN1A, SEM1, and BLM revealed significant relationships and interactions among them, influencing several biological pathways (Figure 6). For example, ATRIP and PCNA were found to share common pathways and interact with genes such as CCNG1, AR, and SP3, suggesting their roles in processes related to the cell cycle, DNA repair, and cellular stress responses. When comparing these findings to genes involved in circadian rhythms, such as AKT1, RPL11, CCNG1, HSPA9, NFKBIA, SP3, CCNA1, MDM4, CDK2, AR, RAD52, CDKN1B, TSG101, KPNA2, PCNA, RAD51AP1, CDKN1A, SEM1, and BLM, notable distinctions arise in their roles within the biological system. The circadian genes primarily regulate biological rhythms and processes related to the cell cycle and metabolism, while the genes identified in this analysis are more connected to DNA repair, cell cycle regulation, and apoptosis. For example, genes like AKT1, MDM4, and RAD52 are crucial in DNA damage response and repair mechanisms, playing roles in regulating the cell cycle and apoptosis. These genes help maintain genomic stability by responding to cellular stress, DNA damage, and genomic instability. On the other hand, CCNG1 and SP3 are primarily involved in transcriptional regulation, indicating a broader role in gene expression and cellular responses to stress. By comparing both sets of genes, it is evident that circadian genes and those involved in DNA repair and apoptosis (AKT1, CDK2, PCNA, RAD51, etc.) intersect in regulating cellular processes, especially in response to DNA damage, oxidative stress, and cell cycle progression. These interactions suggest that genes controlling circadian rhythms might influence or be influenced by mechanisms governing DNA repair, apoptosis, and cell cycle checkpoints, which are crucial for maintaining genomic integrity and cellular homeostasis. These findings highlight the importance of investigating other relevant genes and proteins in future studies.

#### 2.1.5. Gene Expression Analysis

**Figure 7:**
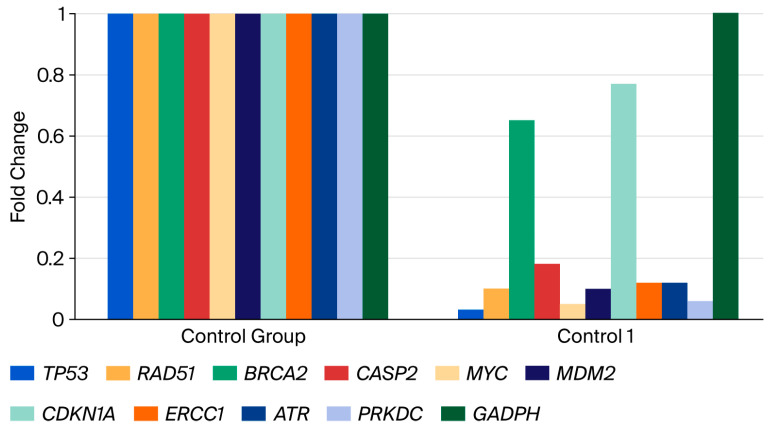
Comparison of expression levels of *Tp53*, *RAD51*, *BRCA2*, *CASP2*, *MYC*, *MDM2*, *CDKN1A*, *ERCC1*, *ATR,* and *PRKDC* genes. (Group 1 = B-134-0).

**Figure 8:**
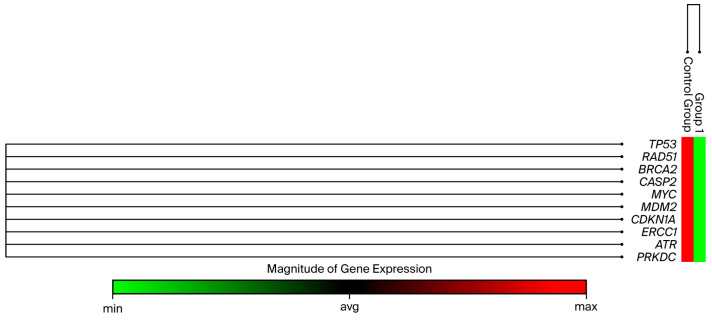
Increasing–decreasing expressions of *Tp53*, *RAD51*, *BRCA2*, *CASP2*, *MYC*, *MDM2*, *CDKN1A*, *ERCC1*, *ATR,* and *PRKDC* genes.

RNA extraction was performed 48 h after administering B-134-0 at determined IC_50_ doses to the SAOS-2 cell line. The extracted RNA was then used to synthesize cDNA, and the expression levels of *Tp53*, *RAD51*, *BRCA2*, *CASP2*, *MYC*, *MDM2*, *CDKN1A*, *ERCC1*, *ATR,* and *PRKDC* genes were analyzed using the ΔΔ_CT_ method. Technical abbreviations have been explained upon first use. Upon evaluation of the results, it was found that the SAOS-2 cell line exhibited significantly reduced gene expression levels compared to the control group. Specifically, *Tp53* gene expression was reduced by 0.03-fold, *RAD51* gene expression by 0.10-fold, *BRCA2* gene expression by 0.65-fold, *CASP2* gene expression by 0.18-fold, *MYC* gene expression by 0.05-fold, *CDKN1A* gene expression by 0.05-fold, and *MDM2* gene expression by 0.10-fold. The observed fold changes for *CDKN1A* gene expression (0.77-fold), *ERCC1* gene expression (0.12-fold), *ATR* gene expression (0.12-fold), and *PRKDC* gene expression (0.06-fold) were not statistically significant (*p* > 0.05), with a 10-fold difference noted. This result is depicted in Figure 7 and Figure 8 and Table 5.

Figure 8 illustrates the relative changes in the expression levels of *TP53*, *RAD51*, *BRCA2*, *CASP2*, *MYC*, *MDM2*, *CDKN1A*, *ERCC1*, *ATR*, and *PRKDC* genes in SAOS-2 cells treated with B-134-0 compared with the untreated control group. The color scale represents the magnitude of gene expression, where green indicates downregulation, red indicates upregulation, and the intensity of the color reflects the degree of deviation from the average expression level. As shown in the figure, most genes, including *TP53*, *RAD51*, *CASP2*, *MYC*, and *MDM2*, exhibited decreased expression, while *BRCA2* and *CDKN1A* displayed relatively higher expression levels, suggesting activation of DNA repair and cell cycle regulatory mechanisms in response to B-134-0 treatment.

#### 2.1.6. DFT and Molecular Docking Analysis

**Figure 9:**
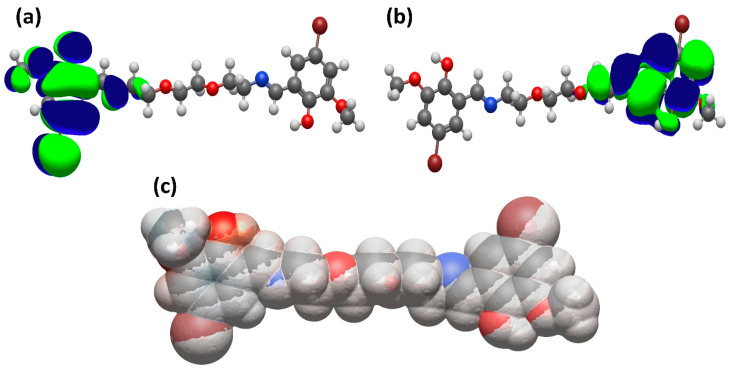
(**a**) HOMO, (**b**) LUMO and (**c**) ESP images of B-134-0 molecule.

Figure 9 visually presents the HOMO, LUMO, and ESP images of the B-134-0 molecule, while Table 6 includes calculated electronic characteristics for same molecular systems. Frontier molecular orbital energies reflect the electron donating and electron accepting ability of the molecules. It is important to note that the molecules with high HOMO energy are good electron donors or Lewis bases. On the other hand, lower LUMO energy values belong to good electron acceptors or Lewis acids in general. One of the most popular parameters used in the analysis of global and local reactivity in Conceptual Density Functional Theory is chemical hardness [28], which was introduced to science as an outcome of the Hard and Soft Acid–Base (HSAB) Principle [29] proposed to predict the products of Lewis acid–base reactions. This concept represents resistance against polarization of the chemical systems, such as atoms, ions, and molecules. The connection between chemical hardness and stability is given through Maximum Hardness Principle (MHP) [30]. According to this principle, chemical hardness, maximized at a steady state, is an important measure of stability. Hard molecules that exhibit high chemical stability have low electron donating abilities. The data presented in Table 6 indicate that the B-134-0 molecule has a relatively high hardness value. First and second electrophilicity indexes are widely used concepts to describe the power of the electrophiles, which gain electrons by reacting with an electron-rich chemical system (nucleophile). The relationship between the electrophilicity index and stability is given via the Minimum Electrophilicity Principle [31], introduced by Chamorro et al. This electronic structure rule states that chemical species with low electrophilicity exhibit high stability. Therefore, the electrophilicity index is a parameter that is minimized at stable states. As a result of the analyses regarding double exchange reactions between inorganic ionic compounds performed by Szentpaly and Kaya [32], it was noted that the second electrophilicity index provides more compatible results with the Minimum Electrophilicity Principle compared to the first electrophilicity index derived by Parr. Both hardness and calculated electrophilicity index values support the idea that the B-134-0 molecule is relatively stable, as noted in the experimental part. Such structural information will guide future studies to obtain more stable and reactive chemical species. For example, if the literature reports that softer molecules with lower chemical hardness interact more strongly with any biological system or gene or that such molecules have higher biological activity, then chemical systems with higher biological activity can be obtained by modifying molecules with higher chemical hardness, including bonded groups that easily pump electrons. In fact, this is a method researchers studying computational molecular design employ before synthesis.

**Figure 10:**
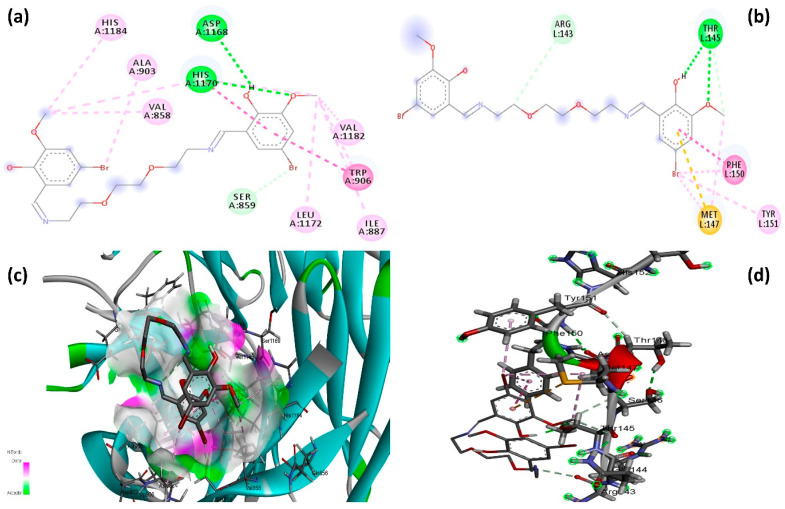
2D and 3D interaction graph of B-134-0 with BRCA2 and CDKN1A proteins. (**a**) 2D interaction graph of B-134-0 with BRCA2. (**b**) 2D interaction graph of B-134-0 with CDKN1A. (**c**) 3D interaction graph of B-134-0 with BRCA2. (**d**) 3D interaction graph of B-134-0 with CDKN1A. (light green: Carbon-Hydrogen bond, green: Hydrogen bond, pink: π-alkyl interaction, purple: π-π interaction, orange: π-sulfur).

**Figure 11:**
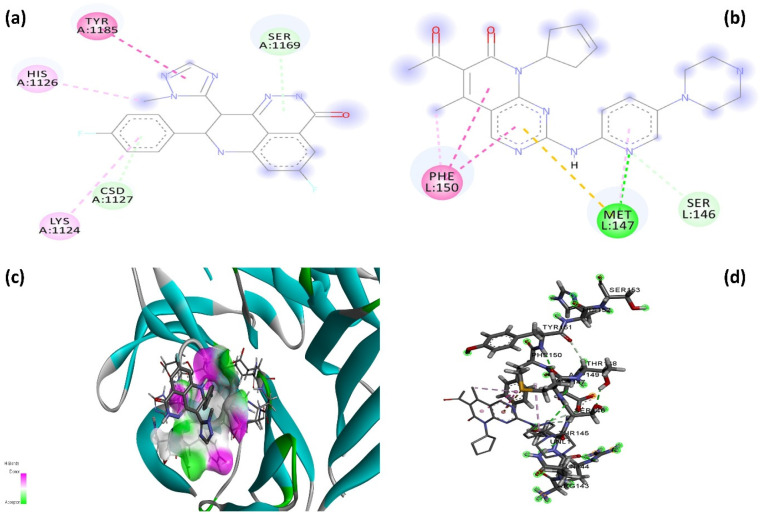
2D and 3D interaction graphs of control molecules (Talazoparib and Palbociclib) with BRCA2 and CDKN1A proteins. (**a**) 2D interaction graph of Talazoparib with BRCA2. (**b**) 2D interaction graph of Palbociclib with CDKN1A. (**c**) 3D interaction graph of Talazoparib with BRCA2. (**d**) 3D interaction graph of Palbociclib with CDKN1A. (light green: Carbon-Hydrogen bond, green: Hydrogen bond, pink: π-alkyl interaction, purple: π-π interaction).

To further assess the interaction potential of B-134-0, molecular docking was carried out using the SwissDock web server against BRCA2 and CDKN1A proteins. The analysis yielded binding energies of −4.743 kcal/mol with BRCA2 and −3.279 kcal/mol with CDKN1A. Figure 10a shows the 2D interaction diagram of the B-134-0 molecule docked into the BRCA2 protein binding pocket, highlighting a comprehensive network of stabilizing interactions. For comparative evaluation, Talazoparib, a clinically approved PARP inhibitor used as a control for BRCA2, exhibited a binding energy of −5.265 kcal/mol, while Palbociclib, a CDK4/6 inhibitor used as a control molecule for CDKN1A, showed a binding energy of −4.979 kcal/mol (Figure 11). These results indicate that although B-134-0 exhibits weaker binding affinity than control inhibitors, comparable docking scores indicate relatively good interaction potential with both BRCA2 and CDKN1A.

B-134-0 is anchored through several hydrogen bonds, hydrophobic contacts, and aromatic interactions that collectively ensure a fit within the binding pocket. Conventional hydrogen bonds are observed with Asp1168 and His1170, residues that play an essential role in stabilizing the molecule through polar interactions. Beyond hydrogen bonding, B-134-0 engages in significant aromatic interactions. π–π stacking and T-shaped interactions with Trp906 and His1170 stabilize the aromatic moieties of B-134-0 within the binding pocket, indicating a favorable orientation driven by π–electron cloud overlap. These aromatic interactions are critical for enhancing the affinity and proper alignment of B-134-0 in relation to the key residues of BRCA2. Hydrophobic interactions also play a major role in the overall stabilization of the complex. Several nonpolar residues, including Val858, Val1182, Leu1172, Ala903, and Ile887, form π–alkyl and alkyl contacts with B-134-0. Such hydrophobic packing interactions not only anchor the molecule within the predominantly binding pocket but also shield it from solvent exposure, which may further strengthen binding stability. Collectively, this interaction profile reveals that the B-134-0 molecule establishes a well-balanced network of polar and non-polar interactions with BRCA2. Figure 10b depicts the 2D interaction profile of the B-134-0 molecule within the binding pocket of the CDKN1A protein. B-134-0 demonstrates a stable binding mode through a combination of hydrogen bonding, π-based interactions, and hydrophobic contacts. Conventional hydrogen bonds are observed with Thr145, while Arg143 contributes via a carbon hydrogen bond, together reinforcing B-134-0’s anchoring within the binding pocket. These polar interactions provide specificity and stabilize the overall orientation of the molecule. In addition to hydrogen bonding, B-134-0 forms π–π stacked and π–alkyl interactions with Phe150 and Tyr151, residues that are frequently involved in stabilizing aromatic systems within the binding pocket. Furthermore, a distinctive π–sulfur interaction with Met147 enhances the overall stabilization of the complex, indicating an important contribution from non-classical but significant van der Waals interactions. Collectively, this interaction network demonstrates that the B-134-0 molecule engages in both polar and hydrophobic/aromatic contacts within the CDKN1A binding pocket.

**Figure 12:**
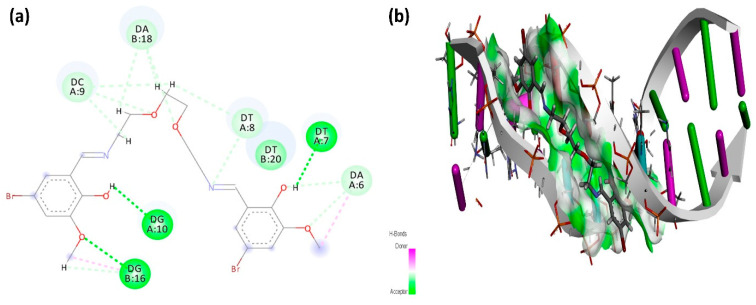
Interaction graphs of B-134-0 with the DNA molecule. (**a**) 2D interaction graph of B-134-0 with the DNA molecule. (**b**) 3D interaction graph of B-134-0 with the DNA molecule. (light green: Carbon-Hydrogen bond, green: Hydrogen bond, pink: π-alkyl interaction).

Molecular docking of the B-134-0 molecule with DNA, performed using PLANTS software, resulted in a binding energy of −124.76 kcal/mol. The B-134-0 molecule’s binding affinity is mediated by a combination of diverse non-covalent forces, including both conventional hydrogen bonding and hydrophobic interaction (Figure 12). The 2D interaction graph reveals that B-134-0 forms carbon hydrogen bonds with nucleobases, such as Thymine8, Adenine18, and Cytosine9. Additionally, conventional hydrogen bond interactions were observed with Guanine10, Guanine16, and Thymine7. The observed π–alkyl interaction with Adenine6 further supports the substantial role of hydrophobic forces in stabilizing the binding mode.

#### 2.1.7. Molecular Dynamics Simulation and Free Energy Calculations

**Figure 13:**
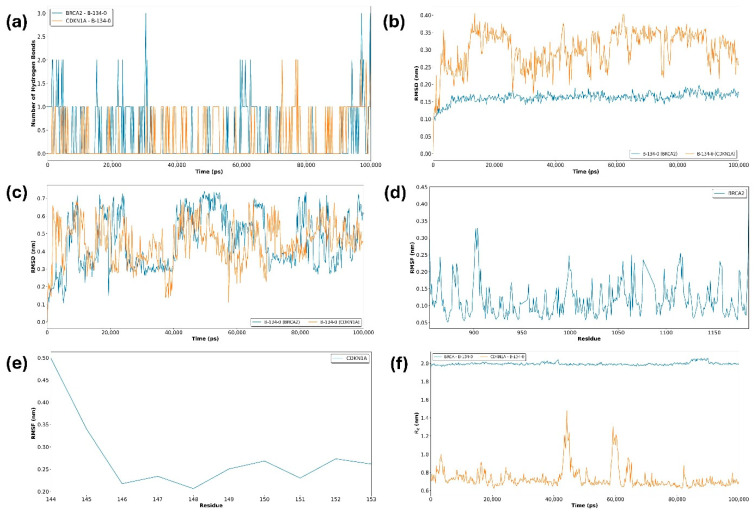
Graphs of data obtained from molecular Dynamics (MD) simulation. (**a**) Hydrogen bond graph of BRCA2 (blue) and CDKN1A (orange) proteins in complex with B-134-0 molecule. (**b**) RMSD graph of BRCA2 (blue) and CDKN1A (orange) proteins. (**c**) RMSD plot of B-134-0 molecule bound to BRCA2 (blue) and CDKN1A (orange) proteins. (**d**) RMSF graph of BRCA2 protein. (**e**) RMSF graph of CDKN1A protein. (**f**) Radius of gyration graph of BRCA2 (blue) and CDKN1A (orange) proteins in complex with B-134-0 molecule.

The hydrogen bond analysis between the B-134-0 molecule and the two target proteins (BRCA2 and CDKN1A) throughout the 100 ns molecular dynamics (MD) simulations revealed distinct interaction patterns. As illustrated in Figure 13a, the BRCA2–B-134-0 complex (blue line) exhibited a more consistent formation of hydrogen bonds compared to the CDKN1A–B-134-0 complex (orange line). The BRCA2–B-134-0 complex occasionally reached up to three hydrogen bonds, with recurrent fluctuations between one and two bonds, indicating a relatively stable hydrogen-bonding profile. In contrast, the CDKN1A complex was characterized by a more transient interaction, with hydrogen bonds appearing intermittently and rarely exceeding two contacts. This suggests that while B-134-0 is capable of forming stabilizing hydrogen bonds with both proteins, the interactions with BRCA2 are more sustained and frequent. Such differences may reflect a stronger binding affinity or a more favorable conformational complementarity of B-134-0 toward BRCA2, which could be relevant in the context of its potential biological activity. The root mean square deviation (RMSD) analysis was performed to assess the structural stability of the BRCA2–B-134-0 and CDKN1A–B-134-0 complexes during the 100 ns molecular dynamics simulation. As shown in Figure 13b, the BRCA2 complex (blue line) exhibited remarkable stability throughout the trajectory, maintaining RMSD values around 0.15–0.18 nm after an initial equilibration phase within the first 10 ns. This indicates that binding of B-134-0 did not induce significant conformational fluctuations in BRCA2, suggesting a stable and well-accommodated molecule interaction within the binding pocket. Conversely, the CDKN1A complex (orange line) displayed higher RMSD values, fluctuating between 0.20 and 0.40 nm across the simulation. Although the system remained stable overall without drastic deviations, the larger amplitude of fluctuations suggests greater flexibility and conformational rearrangements in the CDKN1A–B-134-0 complex. These observations are consistent with the hydrogen bond analysis, supporting the notion that B-134-0 interacts more stably with BRCA2 than with CDKN1A, which may translate into a stronger binding affinity and functional relevance toward BRCA2. Root mean square deviation (RMSD) profiles of the B-134-0 molecule in complex with BRCA2 and CDKN1A proteins over the 100 ns MD simulation are presented in Figure 13c. Both complexes exhibit initial fluctuations during the equilibration phase, after which the RMSD values stabilize within the range of 0.2–0.7 nm, indicating that the protein–ligand systems maintained overall structural stability throughout the simulation. For the BRCA2–B-134-0 complex (blue line), the RMSD curve shows periodic fluctuations but remains consistently centered around 0.3–0.6 nm after 20 ns, suggesting a relatively stable binding mode with minor conformational rearrangements in the binding pocket. In contrast, the CDKN1A–B-134-0 complex (orange line) demonstrates higher dynamic variability, particularly between 10–40 ns and 60–90 ns, where transient increases in RMSD are observed, reflecting conformational adjustments in response to ligand binding. Despite these fluctuations, both trajectories converge toward similar RMSD amplitudes, implying that the ligand preserves stable interactions within the binding pockets of both targets. Overall, the results indicate that the B-134-0 molecule achieves a comparable degree of stability when bound to BRCA2 and CDKN1A, supporting its potential as a dual-targeting candidate. Root mean square fluctuation (RMSF) analysis of the BRCA2–B-134-0 complex (Figure 13d) provides insights into the residue-level flexibility during the 100 ns MD simulation. Overall, the protein exhibited low to moderate fluctuations, with most residues maintaining RMSF values within the 0.05–0.25 nm range, indicating a generally stable backbone conformation. A few regions, notably around residues ~900 and ~1000, displayed slightly elevated fluctuations reaching up to 0.30–0.35 nm, which may correspond to flexible loop regions or solvent-exposed domains. These localized fluctuations are typical in dynamic proteins and are not indicative of global instability but rather reflect the intrinsic mobility of non-structured regions. The overall pattern indicates that the ligand maintains stable interactions with BRCA2 without inducing significant conformational disruption, thereby supporting its potential as a reliable binding partner. The root mean square fluctuation (RMSF) profile of the CDKN1A–B-134-0 complex (Figure 13e) demonstrates limited flexibility across the analyzed residues (144–153), with fluctuations predominantly ranging between 0.20 and 0.35 nm. A higher degree of mobility was observed at the N-terminal end of CDKN1A (residue 144), where the RMSF reached approximately 0.5 nm, suggesting localized flexibility likely associated with a loop or solvent-exposed region. Beyond this initial peak, the fluctuations stabilized at lower values, reflecting restricted motion and a relatively rigid conformation in the binding environment. The consistently low RMSF values across the majority of residues indicate that ligand binding does not induce significant destabilization or large-scale conformational rearrangements within this region of CDKN1A. Instead, the results suggest that B-134-0 stabilizes the local structural environment, supporting the overall stability of the complex during the MD simulation. The radius of gyration (Rg) analysis was performed to evaluate the overall compactness and structural stability of the BRCA2–B-134-0 and CDKN1A–B-134-0 complexes throughout the 100 ns molecular dynamics simulation. As shown in Figure 13f, the BRCA2 complex (blue line) maintained a highly stable Rg value of approximately 2.0 nm across the entire trajectory, indicating that ligand binding did not cause major structural rearrangements or loss of compactness in the protein. This stability is consistent with the RMSD findings, supporting the notion that B-134-0 molecule binding to BRCA2 preserves the protein’s conformational integrity. In contrast, the CDKN1A complex (orange line) displayed lower average Rg values (~0.7–0.9 nm) but exhibited several pronounced fluctuations, particularly around 40–60 ns, where Rg peaked above 1.4 nm. These transient increases suggest conformational flexibility and possible local unfolding events within the CDKN1A complex upon ligand binding. Taken together, the results indicate that B-134-0 maintains the structural compactness of BRCA2 more effectively than CDKN1A, further supporting the higher structural stability and stronger binding propensity of the BRCA2–B-134-0 complex. The binding free energy of the B-134-0 molecule with BRCA2 and CDKN1A was estimated using gmx_MMPBSA, and the results are summarized in Table 7. For both complexes, the dominant stabilizing contribution originated from van der Waals interactions (ΔVDWAALS), with values of −18.34 kJ/mol for BRCA2 and −19.40 kJ/mol for CDKN1A, highlighting the importance of hydrophobic and nonpolar contacts in ligand binding. Electrostatic contributions (ΔEEL) were relatively minor for both systems (−0.68 kJ/mol for BRCA2 and −0.48 kJ/mol for CDKN1A), while polar solvation energy (ΔEGB) acted unfavorably, contributing +1.60 kJ/mol and +1.23 kJ/mol, respectively. The overall gas-phase interaction energies (ΔGGAS) were comparable between the two complexes (−19.02 kJ/mol for BRCA2 and −19.88 kJ/mol for CDKN1A). Similarly, the solvation free energy (ΔGSOLV) provided a small destabilizing contribution, particularly in the CDKN1A complex (−1.61 kJ/mol vs. −0.94 kJ/mol in BRCA2). The total binding free energies (ΔTOTAL) were calculated as −19.96 kJ/mol for BRCA2–B-134-0 and −21.49 kJ/mol for CDKN1A–B-134-0, suggesting that B-134-0 exhibits slightly stronger binding affinity toward CDKN1A. However, when considered together with the RMSD, Rg, and hydrogen bond analyses, it appears that the BRCA2 complex provides a more stable binding environment, whereas the CDKN1A complex, despite its more favorable binding free energy, shows higher conformational flexibility. These findings suggest that the structural stability of the BRCA2 complex may render it a more favorable target for B-134-0 in a biological context.

## 3. Discussion

**Figure 14:**
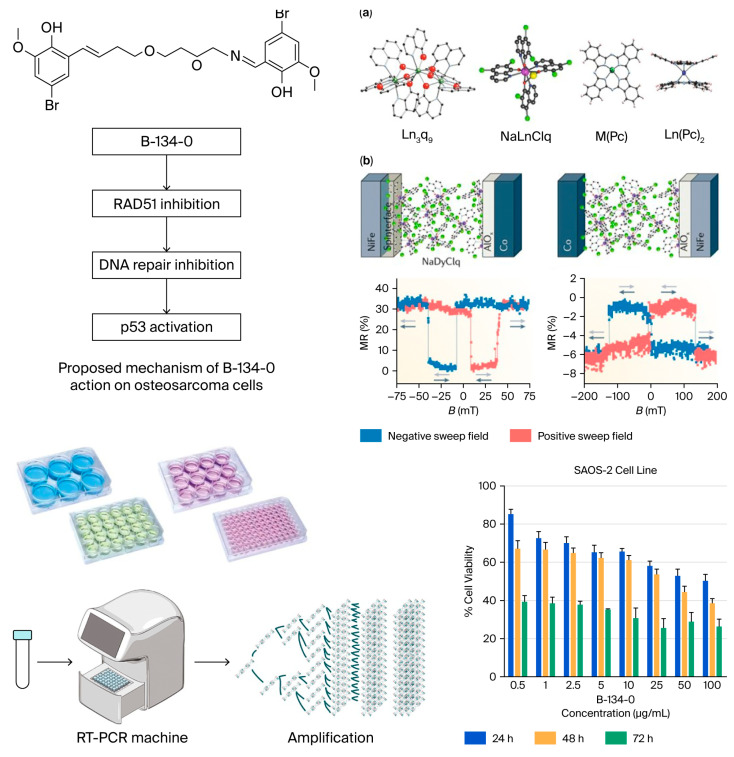
Overview of the experimental workflow and proposed mechanism of B-134-0. (**a**) Molecular structures of the investigated complexes. (**b**) Device schematic and corresponding MR curves under negative and positive field sweeps. The chemical structure and hypothesized mechanism of action of the azomethine compound B-134-0 are illustrated (top left). The compound inhibits *RAD51*, leading to DNA repair suppression and p53 activation in osteosarcoma cells. The schematic also summarizes the experimental workflow, including MTT cytotoxicity assays, RT-qPCR gene expression analysis, and computational modeling studies (DFT and molecular docking), which collectively support the proposed mechanism of B-134-0’s anticancer activity.

The present study investigated the anticancer potential of the newly synthesized azomethine-based compound B-134-0 in SAOS-2 cells; the overall experimental workflow and the proposed mechanism are summarized in Figure 14. Experimental analyses revealed that B-134-0 effectively modulated the expression of several genes associated with apoptosis, cell cycle regulation, and DNA repair. A significant finding was the downregulation of *TP53, RAD51, MYC*, and *MDM2*, accompanied by the upregulation of *CDKN1A* (*p21*) and *BRCA2*. The *TP53* gene encodes a crucial tumor suppressor protein involved in cell cycle arrest, apoptosis, and DNA repair in response to cellular stress [33,34,35]. Reduced *TP53* expression following B-134-0 treatment may alter cellular responses to DNA damage, consistent with earlier studies that identified *TP53* mutations as negative prognostic markers in osteosarcoma [35,36]. Similarly, *RAD51*, a key enzyme in homologous recombination, was significantly downregulated (Figure 14). Previous reports indicate that inhibition of *RAD51* impairs DNA repair and sensitizes cancer cells to chemotherapy and radiation [37,38,39,40,41,42]. The expression of *MYC* was also markedly reduced. Because *MYC* overexpression contributes to tumor progression, resistance to therapy, and apoptosis evasion [43,44,45], its downregulation by B-134-0 implies suppression of oncogenic signaling and reduced proliferative capacity. Likewise, *MDM2*, a negative regulator of p53 associated with metastasis and angiogenesis [46,47,48,49], was downregulated, which could restore p53-mediated growth control and apoptosis induction. Interestingly, *CDKN1A* and *BRCA2* showed higher expression levels compared with other analyzed genes. *CDKN1A* (*p21*), a downstream effector of *p53*, regulates cell cycle arrest during the DNA damage response, while *BRCA2* plays a key role in homologous recombination repair [50,51,52]. This pattern indicates that B-134-0 activates components of the DDR cascade. The significant upregulation of *p53*, *BRCA2*, and *CDKN1A* observed in B-134-0-treated AGS cells collectively suggests activation of a DDR signaling network. As described by Jackson and Bartek (2009) [53], DDR is primarily initiated by the ATM/ATR kinases in response to DNA double-strand breaks or replication stress, leading to phosphorylation of checkpoint kinases (CHK1 and CHK2), stabilization of *p53*, and subsequent induction of *CDKN1A* (*p21*). This checkpoint activation temporarily halts cell cycle progression, enabling DNA repair. Concurrently, elevated *BRCA2* expression suggests engagement of homologous recombination repair as part of the p53–p21 regulatory axis. Supporting this interpretation, Fridlich et al. (2015) [54] demonstrated that the BRCA1/2 pathway is essential for repairing oxidative or replication-associated DNA lesions that persist into the S phase and transform into double-strand breaks. Therefore, the molecular data collectively indicate that B-134-0 induces genotoxic and oxidative stress and activates an integrated ATM/ATR–CHK–p53–p21–BRCA2 signaling pathway that coordinates DNA damage sensing, checkpoint control, and high-fidelity repair [53,54]. This mechanistic insight may explain the observed cytotoxic and growth-inhibitory effects of B-134-0, suggesting that its anticancer activity arises from DDR-mediated genomic surveillance and apoptosis induction. In addition, *ERCC1*, *ATR*, and *PRKDC* genes were downregulated following treatment. These genes are associated with nucleotide excision repair and replication checkpoint control, and their suppression may enhance apoptotic susceptibility [55,56,57]. Molecular docking and MD simulations further supported the experimental data. B-134-0 exhibited high binding affinity to BRCA2 (−4.743 kcal/mol) and CDKN1A (−3.279 kcal/mol) proteins. Stable hydrogen bonds, π–π stacking, and hydrophobic interactions were detected in both complexes, with the BRCA2 complex showing greater structural stability during MD analysis. These results are consistent with experimental findings, indicating that B-134-0 directly interacts with DNA repair and cell cycle regulatory proteins.

Collectively, the experimental and computational results suggest that B-134-0 exerts its anticancer effects through a dual-targeting mechanism involving BRCA2 and CDKN1A. By simultaneously impairing DNA repair (through RAD51 and BRCA2 modulation) and enhancing checkpoint signaling (via the p53–p21 axis), B-134-0 induces apoptosis and inhibits proliferation in osteosarcoma cells. These findings position B-134-0 as a promising candidate for targeted osteosarcoma therapy, potentially offering enhanced efficacy and reduced systemic toxicity compared with current chemotherapeutic agents.

Molar Diamagnetic Susceptibility of B-134-0 molecule from Property Prediction from Structural Differences (PPFSD) Method

Biological activity of the molecules is closely related to the chemical reactivity of the molecular systems. In addition to the relationship with the chemical reactivity of the parameters, such as hardness, polarizability, and electrophilicity, magnetic properties have also been correlated with reactivity within the CDFT [58,59].

The relationship with the stability of the magnetizability is given through the Minimum Magnetizability Principle introduced by Chattaraj and coworkers [60]. This principle implies that magnetizability is minimized in stable states like polarizability. So, it can be said that molecules with high reactivity and biological activity should have low magnetizability. Because magnetizability is a parameter related to stability, we also calculated the molar diamagnetic susceptibility for our newly synthesized molecule using the Property Prediction from Structural Differences (PPFSD) method, a method developed by one of our authors. This method developed by Kaya [61] is one of the fastest alternative methods used for the estimation of the molar diamagnetic susceptibility of the organic molecules. In the first stage of the method, the contribution of the hydrogen atom to molar diamagnetic susceptibility is determined by the equation given below.

(1) $$(-\chi_{m}(-H))\;=\;(-\chi_{m}(CH_{4}))\;-\;\frac{\left(-\chi_{m}(C_{2}H_{6})\right)}{2}$$

here, $\chi_{m}(CH_{4})$ and $\chi_{m}(C_{2}H_{6})$ stand for the molar diamagnetic susceptibilities of methane and ethane molecules, respectively. Experimentally determined molar diamagnetic susceptibility values for methane and ethane molecules are 17.4 × 10^−6^ and 26.8 × 10^−6^ cm^3^/mol, respectively. From these values, the contribution of the hydrogen atom is approximately determined as 4.0 × 10^−6^ cm^3^/mol. This determined value is used for the estimation of the contribution of other functional groups. In the original article introducing the PPFSD method and in another article covering the application of this method to amino acids, Kaya [62] reported functional group contributions in detail. The functional groups seen in the structure of the studied molecule, the symbols representing these groups, and the group contribution values determined for the groups are presented in detail in Table 8. In the structure of our molecule, two -OCH_3_, two -Br, two -OH, two C=NH, two phenyls, six -CH_2_-, and two -O- groups appear. Of these groups, only the contribution of the -O- group has not been reported in our previous articles. The contribution of this group, represented by the symbol Φ_17_, has been calculated using the experimental molar diamagnetic susceptibility value of the diethyl ether (CH_3_CH_2_OCH_2_CH_3_) molecule as follows and added to the relevant table.

(2) $$(-\chi_{m}(B-134-0))=2F+6I+2J+2P+2\Delta +2\Phi_{17}-8A$$

(3) $$(-\chi_{m}(-O-))=55.10-(2\;\times \;13.4)-(2\;\times \;11.8)=4.7{\;cm}^{3}/mol$$

From the given group contribution values, we can design the following calculation scheme for the prediction of the molar diamagnetic susceptibility of the B-134-0 molecule:

(4) $$(-\chi_{m}(B-134-0))=2F+6I+2J+2P+2\Delta +2Þ+2\Phi_{17}-8A$$

With the help of this scheme, the molar diamagnetic susceptibility of the B-134-0 molecule is first predicted as −282.78 × 10^−6^ cm^3^/mol.

## 4. Materials and Methods

### 4.1. Synthesis Procedure

The compound was synthesized by condensing an aldehyde with an amine. To do this, 0.1 mmol of 5-bromo-2-hydroxy-3-methoxybenzaldehyde was dissolved in 5 mL of ethanol, and then 0.2 mmol of 2,2′-(ethane-1,2-diylbis(oxy))bis(ethan-1-amine) was added. The reaction mixture was stirred at room temperature for 2.5 h. At the end of the reaction time, the mixture was poured onto ice. The resulting precipitate was filtered, washed with distilled water, and dried at room temperature. The yield of the compound is 85–90% with a melting point of 135–136 °C. The ^1^H NMR spectrum is as follows: (DMSO-d6, δ, ppm) 3.52 s (4H, 2NCH_2_), 3.63–3.68 m (8H, 3OCH_2_), 3.76 s (6H, 2OCH_3_), 7.04–7.16 m (4H, 2Ar), and 8.41 s (2H, 2CHN). The 13C NMR spectrum: (DMSO-d6, δ, ppm) 56.40 (2NCH_2_), 56.49 (2OCH_2_), 69.90 (2OCH_2_), 70.13 (2OCH_3_), 107. 24 (2C, Ar), 117.16 (2CH, Ar), 118.67 (2C, Ar), 125.23 (2CH, Ar), 150.49 (2C, Ar), 155.03 (2C, Ar), and 166.13 (2NCH) (Figure 1).

### 4.2. Cell Culture

SAOS-2 (ATCC HTB-85) is a cell line with epithelial morphology isolated from the mouth of an 11-year-old Caucasian osteosarcoma patient. The patient was treated with a combination of RTG, methotrexate, adriamycin, vincristine, cytoxan, and aramycin C. This cell line is part of a large series of human tumor lines isolated and characterized by J. Fogh and G. Trempe (www.atcc.org). SAOS-2 osteosarcoma cells were cultured in Dulbecco’s Modified Eagle’s Medium (DMEM) supplemented with 100 Units/mL of penicillin and 10% fetal bovine serum (FBS) at 37 °C with 5% CO_2_. The cells were subcultured upon reaching confluency.

### 4.3. In Vitro Cytotoxicity Determination (MTT)

SAOS-2 osteosarcoma cancer cells were cultured at a density of 75. The cells were then treated with 2 mL of Trypsin/EDTA and incubated in a 5% CO_2_ oven before being separated from the culture surface. The SAOS-2 cell line was seeded at a density of 1 × 10^5^ cells per well in 96-well plates and exposed to various concentrations of B-134-0 (ranging from 100 to 0.5 μg/mL) for 24, 48, and 72 h. The anti-cancer potential of B-134-0 in the SAOS-2 cell line was evaluated using the MTT assay. A dosage of 10 µL of the supplement was administered in each well. Then, MTT was aspirated, and 100 μL of DMSO was added. The mixture was left for 15 min at room temperature. Subsequently, absorbance measurements were obtained at 570 nm using GraphPad Prism7 software to determine benchmark IC_50_ values.

### 4.4. Cell Morphology

SAOS-2 osteosarcoma cancer cells (5 × 10^5^ cells per well) were cultured and treated with 1 μM of compound B-134-0 per cell. The resulting alterations in cellular morphology were subsequently assessed using a 20× magnification cell imaging device (ZEISS Axio Vert.A1).

### 4.5. Bioinformatics Analysis

The protein–protein interactions with proteins involved in the cell cycle and the apoptosis pathway were determined using the STRING v11 program (https://string-db.org/). Gene–gene interactions (GGI) were analyzed using GenMANIA software (https://genemania.org/, accessed on 1 December 2025).

### 4.6. RNA Isolation from Cell Culture Samples

The IC_50_ concentrations of each sample were determined after 48 h of incubation. Once the SAOS-2 cells reached the desired growth rate, they were seeded in six-well plates. Subsequently, RNA isolation from the SAOS-2 cell line was performed following the RNeasy Plus Mini kit protocol.

### 4.7. cDNA Synthesis

The cDNA was synthesized from the RNAs according to the kit protocol.

### 4.8. Quantitative Polymerase Chain Reaction (qPCR) Analysis

The levels of expression for *Tp53*, *RAD51*, *BRCA2*, *CASP2*, *MYC*, *MDM2*, *CDKN1A*, *ERCC1*, *ATR*, and *PRKDC* genes were identified using the RT-PCR device in conjunction with the optimized RT^2^ SYBRGreen qPCR Mastermix kit. *GAPDH* was used as the housekeeping gene and internal control to assess expression level differences between the control and experimental groups. The fluorescent dye SYBRGreen was used, and 25 µL of the qPCR mixture was prepared according to the kit protocol from the cDNA-containing samples. The statistical analysis of the results was performed using the ΔΔ^CT^ method with software provided by https://dataanalysis2.qiagen.com/pcr (accessed on 1 December 2025).

### 4.9. Statistical Analysis

In our cell culture study, the sample sizes were optimized through statistical power analysis. Using GraphPad Prism 8.0.1 software, test types (one-way ANOVA and Student’s *t*-test), Cohen’s d (0.5, medium effect) derived from pilot data, 80% statistical power, and a 5% significance level were applied. Based on the power analysis, a minimum of 8 biological replicates per group was determined, and the experiments were designed accordingly. For data analysis, gene expression data were normalized using the ΔΔ_Ct_ method. Differences between two groups were analyzed using Student’s *t*-test, while multiple group comparisons were performed using one-way ANOVA. Appropriate post hoc tests were applied to determine significant differences. Normality (Shapiro–Wilk test) and homogeneity of variance (Levene test) were assessed to confirm the suitability of the statistical tests (Figure 3, Table 3). To minimize bias and variability, treatment groups were randomly assigned, with at least three technical replicates for each biological replicate. Biological diversity was ensured by using cells from different passages. Variability between the data was carefully analyzed and reported graphically.

### 4.10. Computational Details

In addition to experimental studies, DFT-based calculations can also be used to quickly gain insight into the reactive or stable nature of newly synthesized compounds. For this purpose, the reactivity descriptors and related electronic structure principles proposed within the framework of Conceptual Density Functional Theory, which is the branch of DFT related to reactivity analysis, are frequently used by theoretical and computational chemists. Here, CDFT calculations for the B-134-0 molecule were performed with the help of the B3LYP-def2-SVP level of the theory and using ORCA software (version 4.0.). CDFT presents the following simplified mathematical relations for chemical potential (µ), electronegativity (χ), hardness (η), and softness (σ) [63,64].

(5) $$\mu =-\chi =\;{\left[\frac{\partial E}{\partial N}\right]}_{\nu (r)}=\;-\;\left[\frac{I+A}{2}\right]$$

(6) $$\eta =\;{\left[\frac{\partial \mu }{\partial N}\right]}_{\;\nu (r)}=\;{\left[\frac{\partial^{2}E}{\partial N^{2}}\right]}_{\;\nu (r)}=I-A$$

(7) σ=1/η

where E and N represent the total electronic energy and the total number of the electrons, respectively. I and A stand for the ionization energy and the electron affinity of the studied chemical system, respectively.

First (ω_1_) and second (ω_2_) electrophilicity indexes used to predict electrophilic and nucleophilic behaviors of the molecules are calculated as follows [65,66]:

(8) $$\omega_{∥}=\chi^{2}/2\eta =\mu^{2}/2\eta ={\left(I+A\right)}^{2}/8(I-A)$$

(9) $$\omega_{2}=I.A/(I-A)$$

The ability of a molecule to donate and accept electrons is crucial for its interaction with a biological system. These properties play a decisive role in the strength of the interaction. To estimate the electrodonating and electroaccepting powers of the chemical systems, the formulae derived by Gazquez et al. are given as [67]

(10) $$\omega^{-}={\left(3I+A\right)}^{2}/\left(16(I-A)\right)$$

(11) $$\omega^{+}={\left(I+3A\right)}^{2}/\left(16(I-A)\right)$$

Net electrophilicity (Δω±) introduced by Chattaraj [68] is calculated as

(12) $$\Delta \omega \pm =\omega^{+}+\omega^{-}$$

In the present paper, the ionization energy and the electron affinity of the studied molecule were predicted using Koopmans Theorem [69], providing frontier orbital energy-based relations given below.

(13) $$I=-E_{HOMO}$$

(14) $$A=-E_{LUMO}$$

EHOMO and ELUMO are the energies of HOMO and LUMO orbitals of the molecule, respectively.

### 4.11. Molecular Docking Analysis

After geometry optimization with DFT, the energetically stable structure of the B-134-0 molecule was used for molecular docking studies through the SwissDock web server [70]. Three-dimensional structures of BRCA2 (PDB ID: 3EU7) and CDKN1A (PDB ID: 2ZVW), which are highly expressed proteins in gene expression data (Figure 7), were retrieved from the RCSB Protein Data Bank [71]. In the molecular docking analysis, Talazoparib (PubChem ID: 135565082), a clinically approved PARP inhibitor used in patients carrying BRCA mutations, was employed as the reference molecule for the BRCA2 protein. Similarly, Palbociclib (Pub-Chem ID: 5330286), a selective CDK inhibitor, was used as the reference molecule for the CDKN1A protein. Additionally, molecular docking was performed to determine the interaction of the B-134-0 molecule with DNA (PDB ID: 1BNA). PLANTS software [72] was used for this analysis. Protein preparation was performed in UCSF Chimera [73] using the Dock Prep module, which included removal of non-standard residues and water molecules, addition of hydrogen atoms, and assignment of partial charges. The DFT-optimized molecular structure was then converted into the required input format (.mol2) for docking simulations. The resulting protein–ligand complexes were further examined in BIOVIA Discovery Studio Visualizer [74], where two-dimensional interaction diagrams were generated to highlight hydrogen bonds, hydrophobic contacts, π–π stacking, and other significant non-covalent interactions within the binding pocket.

BRCA2 and CDKN1A proteins were selected for DFT and molecular docking analyses as they are key downstream effectors of the p53-mediated DDR pathway, representing essential regulators of DNA repair and cell cycle arrest.

### 4.12. Molecular Dynamics Simulation and Free Energy Calculations

Molecular dynamics (MD) simulations were performed using GROMACS software (version 2024.1) [75] to investigate the structural stability and dynamic behavior of the docked complexes. The top-ranked docking poses of the B-134-0 molecule with BRCA2 and CDKN1A were selected as the initial structures. The CHARMM27 force field [76] was applied to parameterize the protein, while the ligand topology and parameters were generated using the SwissParam server [77]. Each protein–ligand complex was placed in a dodecahedral box with at least 1.0 nm padding from the solute, solvated with TIP3P water molecules, and neutralized by adding appropriate counterions. Prior to the production simulations, all systems were subjected to energy minimization via the steepest descent algorithm to eliminate steric clashes. Equilibration was then carried out in two stages, with 250 ps under the NVT ensemble (constant number of particles, volume, and temperature) followed by 250 ps under the NPT ensemble (constant number of particles, pressure, and temperature), during which positional restraints were applied to heavy atoms. Temperature was maintained at 300 K using the V-rescale thermostat, while pressure was controlled at 1 bar with the Parrinello–Rahman barostat. Long-range electrostatics were calculated using the Particle Mesh Ewald (PME) method with a cutoff of 1.4 nm for Coulomb and van der Waals interactions. Following equilibration, 100 ns production MD simulations were conducted for each complex without restraints. Trajectory data were analyzed to assess structural stability and flexibility in terms of root mean square deviation (RMSD), root mean square fluctuation (RMSF), and radius of gyration (Rg) using standard GROMACS utilities. To quantify the binding strength of the protein–ligand complexes, binding free energy calculations were carried out using the gmx_MMPBSA tool [78], which implements the Molecular Mechanics/Poisson–Boltzmann Surface Area (MM-PBSA) approach. Binding free energies (ΔG_bind) were estimated from snapshots obtained from the entire simulation. Energy decomposition analyses were also performed to evaluate the contributions of van der Waals, electrostatic, polar solvation, and non-polar solvation components to the overall binding affinity.

## 5. Conclusions

To conclude, the newly synthesized molecule (B-134-0) exhibited strong anticancer effect on SAOS-2 cancer cells and regulated the expression of certain genes involved in processes such as cell cycle and apoptosis. The investigation of the interaction between our new molecule and *BRCA2* and *CDKN1A* genes showed that B-134-0 has high binding affinity to these biological systems. Calculated CDFT-based reactivity descriptors, such as hardness, electronegativity, and electrophilicity, imply that this new molecule has relatively high stability. According to both experimental and theoretical analyses, it should be noted that this new molecule can be considered as an alternative medicine or drug additive in the treatment of osteosarcoma. Molar diamagnetic susceptibility of the molecule was reported using the Property Prediction from Structural Differences method.

## 6. Limitations

This study has several limitations. It was conducted using a single SAOS-2, which may not fully reflect the biological heterogeneity of osteosarcoma. Apoptosis assays and protein-level validations, such as Western blot or caspase activity analyses, were not performed, and in vivo experiments were not included due to budgetary and infrastructural constraints. Nevertheless, the findings offer important preliminary insights into the molecular effects of B-134-0, and ongoing studies by our group using another azomethine-based compound at the gene and clinical levels aim to further validate and expand these results.

## Figures and Tables

**Table 1:** Comparison of the effect of B-134-0 compound on SAOS-2 cell viability at concentrations of 100–0.5 µg/mL after 24, 48, and 72 h of incubation (Mean ± 95% CI, *p* value).

	Mean	95.00% CI	*p* Value
24 h vs. 48 h	64.96	−4.336 to 19.84	0.2609 ^a^
24 h vs. 72 h	64.96	20.34 to 44.52	*p* < 0.0001 **** ^a^
48 h vs. 72 h	57.20	12.59 to 36.76	0.0001 *** ^a^

^a^ One-way ANOVA with Tukey’s multiple comparisons test. Significance indicates (**** *p* < 0.0001, *** 0.0001).

**Table 2:** Comparison of IC_50_ values of B-134-0 on the SAOS-2 cell line after 24, 48, and 72 h of incubation.

IC_50_ (μg/mL)			
Incubation Period	24 h	48 h	72 h
B-134-0	71.58 μg/mL	54.36 μg/mL	12.59 μg/mL

IC_50_: Half-maximal inhibitory concentration.

**Table 3:** Shapiro–Wilk and Kolmogorov–Smirnov test values for normal distribution in SAOS-2 cell line.

Anderson–Darling Test			
A2	0.1958	0.5140	0.4109
*p* value	0.8324	0.1314	0.2556
Passed normality test (alpha = 0.05)?	Yes	Yes	Yes
*p* value	ns	ns	ns
D’Agostino and Pearson test			
K2	0.3880	1.741	3.316
*p* value	0.8236	0.4188	0.1905
Passed normality test (alpha = 0.05)?	Yes	Yes	Yes
*p* value	ns	ns	ns
Shapiro–Wilk test			
W	0.9635	0.8588	0.8808
*p* value	0.8429	0.1167	0.1917
Passed normality test (alpha = 0.05)?	Yes	Yes	Yes
*p* value	ns	ns	ns
Kolmogorov–Smirnov test			
KS distance	0.1381	0.2554	0.1932
*p* value	>0.1000	>0.1000	>0.1000
Passed normality test (alpha = 0.05)?	Yes	Yes	Yes
*p* value	ns	ns	ns

Significance indicates (*p* > 0.05).

**Table 4:** Protein–protein interactions and significance values.

Protein	Related Protein	Homology Score
BRCA2	RAD51	0.999
CCNA1	CDKN1A	0.999
CCND1	CDK4	0.999
CCND1	CDKN1A	0.999
CDK4	CDKN1A	0.999
CDKN1A	TP53	0.999
MDM2	TP53	0.999
MYC	TP53	0.997
PRKDC	TP53	0.997
ATR	TP53	0.996
RAD51	RPA1	0.996
BRCA2	TP53	0.995
CCNA1	CDK4	0.988
ATR	RPA1	0.983
CDKN1A	MYC	0.981
CDKN1A	MDM2	0.980
CCNA1	TP53	0.974
CCND1	MYC	0.969
CDK4	MYC	0.966
CDK4	TP53	0.966
ERCC1	PRKDC	0.958
CCND1	TP53	0.946
BRCA2	CDK4	0.943
ATR	RAD51	0.942
CCND1	MDM2	0.926
CDK4	RAD51	0.913
ERCC1	RAD51	0.908
ERCC1	RPA1	0.898
BRCA2	PRKDC	0.887
CDK4	MDM2	0.884
BRCA2	ERCC1	0.877
BRCA2	CCND1	0.861
MYC	SIRT1	0.860
PRKDC	RPA1	0.840
ATR	BRCA2	0.831
PRKDC	RAD51	0.823
MDM2	MYC	0.813
CCNA1	MDM2	0.810
BRCA2	RPA1	0.807
CASP2	TP53	0.794

**Table 5:** Average CT and fold change values of cell cycle and apoptosis genes.

Gene	Average CT Control	Average CT B-134-0	Fold Change
Tp53	32.49	32.73	0.03
RAD51	32.99	31.49	0.10
BRCA2	25.49	21.36	0.65
CASP2	30.01	27.71	0.18
MYC	30.70	30.14	0.05
MDM2	30.39	28.90	0.10
CDKN1A	32.75	28.37	0.77
ERCC1	30.29	28.59	0.12
ATR	29.95	28.30	0.12
PRKDC	31.17	30.43	0.06
GADPH	24.93	20.17	1.00

Significance indicates (*p* > 0.05). *GAPDH*: Glyceraldehyde 3-phosphate dehydrogenase. CT: cycle threshold.

**Table 6:** Calculated electronic characteristics of the B-134-0 molecule.

EHOMO (eV)	−6.899
ELUMO (eV)	−1.627
Ionization energy (I)	6.899
Electron affinity (A)	1.627
χ	4.273
µ	−4.273
η	5.272
σ	0.189
ω_1_	1.723
ω_2_	2.129
ω−	5.908
ω+	1.645
Δω±	7.553

**Table 7:** Free energy components of BRCA2 and CDKN1A proteins in complex with B-134-0 molecule.

Energy Component	BRCA2–B-134-0	CDKN1A–B-134-0
ΔVDWAALS (kJ/mol)	−18.34	−19.40
ΔEEL (kJ/mol)	−0.68	−0.48
ΔEGB (kJ/mol)	1.60	1.23
ΔGGAS (kJ/mol)	−19.02	−19.88
ΔGSOLV (kJ/mol)	−0.94	−1.61
ΔTOTAL (kJ/mol)	−19.96	−21.49

**Table 8:** Determined group contribution values for the prediction of the molar diamagnetic susceptibility of organic structures.

Group	Symbol	Determined Group Contribution Value × 10^6^ (cm^3^/mol)
-H	A	4.0
-Br	F	26.65
-CH_2_- (in chain)	I	11.8
-OH	J	10.8
-Phenyl	P	50.84
-(C=NH)	Δ	11.1
-OCH_3_	Þ	17.9
-O-	Φ17	4.7

## Data Availability

The original contributions presented in this study are included in the article. Further inquiries can be directed to the corresponding author.

## Abbreviations

The following abbreviations are used in this manuscript:

MCF-7	Breast cancer
CASP2	Caspase 2
HCT116	Colorectal cancer
CDKN1A	Cyclin dependent kinase inhibitor 1A
BRCA2	DNA repair associated
GAPDH	Glucose-6-Phosphate Dehydrogenase
HepG2	Liver cancer
A549	Lung cancer
SAOS-2	Osteosarcoma cell line
PRKDC	Protein kinase
ATR	Rad3-related kinase
RAD51	RAD51 recombinase
ERCC1	The Excision Repair Cross Complementation Group 1
TP53	Tumor protein p53
MDM2	Ubiquitin-protein ligase
